# Evaluation of the metformin effects on Anti-Müllerian Hormone in women with polycystic ovarian syndrome: A double-blind randomized clinical trial

**DOI:** 10.18502/ijrm.v17i2.3992

**Published:** 2019-03-20

**Authors:** Masoud Rahmanian

##  Dear Editor,

Anti-Müllerian hormone (AMH) is made by the granulosa cells of preantral and small antral follicles which blocks the transition from the primordial to the primary follicular stage. Metformin may be associated with a decrease in AMH serum level and antral follicles in women who suffer from polycystic ovary syndrome (PCOs)

It was reported that metformin in women with polycystic ovary syndrome (PCOs) is associated with a decrease in both AMH serum level and antral follicles.

The objective of the current letter was the evaluation of the effects of metformin on hormonal profile of women with PCOs. In this prospective, randomized, double-blind controlled clinical trial, 42 women (aged 17–45 yr) with PCOs who were randomly allocated to receive 500 mg Metformin orally three times a day or placebo for three months were included. Fasting plasma glucose, follicular stimulating hormone, luteinizing hormone (LH), prolactin (PRL), testosterone and AMH levels were measured at baseline and at the end of the period. In this study, independent and paired *t*-test were used for quantitative comparison and chi-square analysis for qualitative variables. Logistic regression analysis was done to identify independent risk factors, and P-value of lower than 0.05 were considered significant. All of the statistical analyses were done by SPSS software, version 20.0. We didn't find any significant change after the study between treatment and control groups in hormonal profiles especially AMH. However, in subgroup analysis, we revealed that AMH and LH levels decreased significantly in normal weight patients (p= 0.024, 0.048, respectively) and prolactin levels in subgroup of overweight patients (p= 0.001). Moreover, patients in metformin group at the end of study had more regular menses, more weight loss, and lower hair loss (p= 0.001, 0.04, 0.014, respectively). Women with PCOs have elevated levels of LH that is secondary to increased sensitivity of pituitary to GnRH. Increased levels of LH lead to hyperandrogenism. Metformin can improve this condition. In this study, we observed that LH was decreased significantly in the metformin group of PCOs patients (p= 0.05). It is in agreement with some of the previous studies, however, De Leo and *et al*. reported a non-significant decrease in LH levels after treatment with metformin (1). Pieces of evidence showed that AMH levels in PCOs patients are 2 to 3 times higher than age-matched normal women (2), and this condition is a marker for PCOs and can be a surrogate test of hyperandrogenism (3). Metformin in our study improved the irregularity in the menstrual cycle (p< 0.001), hair loss (p= 0.014), and prolactin secretion (p= 0.024), as well as LH secretion (p= 0.05); however, it didn't show any significant decrease on AMH levels. Metformin can reduce ovarian volume in PCOs patients especially in hyperinsulinemic subgroups (4); however, we observed that in normal weight subgroup of patients, metformin decreased AMH significantly (p= 0.024), and in overweight or obese patients, prolactin decreased in response to metformin, significantly (p= 0.001). Moreover, metformin leads to improvement in follicular development with fewer percentage of preantral follicles and cysts and higher percentages of antral follicles (5). Considering these pieces of evidence, it seems that AMH after metformin treatment in PCOs patients was secret in comparable amounts of pretreatment; however, the source of secretion after starting metformin is different from pretreatment and switched from small follicles and cysts to more developed follicles and structures such as antral follicles and corpora lutea. Further investigations that can differentiate secreted AMH from a different source will shed more light on this field.

The most important limitation of our study is the small sample size. GI side effects of metformin caused some patients in metformin group to refuse getting the treatment. Studies with more attendants can display the difference between different phenotypes of PCOs patients in response to metformin. In conclusion, this study showed that metformin can cause favorable effects on the hormonal profile of PCOs patients, and although AMH levels were not decreased significantly, menstruation improved in the treated group.

**Jam Ashkezari S M.Sc., Nasim Namiranian M.D., Somaye Gholami M.Sc., Maryam Elahi M.D., Masoud Rahmanian M.D.**

Diabetes Research Center, Shahid Sadoughi University of Medical Sciences, Yazd, Iran.
